# Poly[[diaqua­(1,10-phenanthroline-κ^2^ *N*,*N*′)(μ_3_-4-sulfonato­benzene-1,2-di­car­boxyl­ato-κ^4^ *O* ^1^:*O* ^2^,*O* ^2′^:*O* ^4^)erbium(III)] dihydrate]

**DOI:** 10.1107/S1600536812003467

**Published:** 2012-01-31

**Authors:** Kou-Lin Zhang, Jian-Guo Lin, Seik Weng Ng

**Affiliations:** a College of Chemistry and Chemical Engineering, Yangzhou University, Yangzhou 225002, People’s Republic of China; b Department of Chemistry, University of Malaya, 50603 Kuala Lumpur, Malaysia; c Chemistry Department, King Abdulaziz University, PO Box 80203 Jeddah, Saudi Arabia

## Abstract

The 4-sulfophthalate trianion in the polymeric complex, {[Er(C_8_H_3_O_7_S)(C_12_H_8_N_2_)(H_2_O)_2_]·2H_2_O}_*n*_, bridges three water/phenanthroline-coordinated Er^III^ ions to form a three-dimensional network architecture. The metal atom is further chelated by a carboxyl­ate group and is covalently bonded to a monodentate carboxyl­ate group as well as to a monodentate sulfonate group in a distorted square anti­prismatic geometry. The coordinating water molecules and the lattice water molecules, one of which is disordered over two positions [major component 65 (3)%], are hydrogen bonded to the network.

## Related literature

For a related aqua­(1,10-phenanthroline)Eu^III^ derivative, see: Xiao *et al.* (2010 ▶).
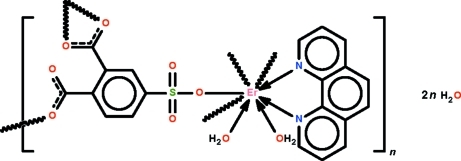


## Experimental

### 

#### Crystal data

[Er(C_8_H_3_O_7_S)(C_12_H_8_N_2_)(H_2_O)_2_]·2H_2_O
*M* *_r_* = 662.69
Monoclinic, 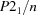

*a* = 14.3924 (1) Å
*b* = 9.6206 (2) Å
*c* = 17.4245 (3) Å
β = 105.840 (1)°
*V* = 2321.04 (6) Å^3^
*Z* = 4
Mo *K*α radiation
μ = 3.77 mm^−1^
*T* = 293 K
0.50 × 0.20 × 0.20 mm

#### Data collection

Bruker SMART APEX diffractometer
Absorption correction: multi-scan (*SADABS*; Sheldrick, 1996 ▶) *T*_min_ = 0.500, *T*_max_ = 1.000
7081 measured reflections
4014 independent reflections
3799 reflections with *I* > 2σ(*I*)
*R*_int_ = 0.023

#### Refinement

*R*[*F* ^2^ > 2σ(*F* ^2^)] = 0.037
*wR*(*F* ^2^) = 0.095
*S* = 1.09
4014 reflections
353 parameters
33 restraints
H atoms treated by a mixture of independent and constrained refinement
Δρ_max_ = 0.97 e Å^−3^
Δρ_min_ = −1.21 e Å^−3^

### 

Data collection: *APEX2* (Bruker, 2005 ▶); cell refinement: *SAINT* (Bruker, 2005 ▶); data reduction: *SAINT*; program(s) used to solve structure: *SHELXS97* (Sheldrick, 2008 ▶); program(s) used to refine structure: *SHELXL97* (Sheldrick, 2008 ▶); molecular graphics: *X-SEED* (Barbour, 2001 ▶); software used to prepare material for publication: *publCIF* (Westrip, 2010 ▶).

## Supplementary Material

Crystal structure: contains datablock(s) global, I. DOI: 10.1107/S1600536812003467/xu5455sup1.cif

Structure factors: contains datablock(s) I. DOI: 10.1107/S1600536812003467/xu5455Isup2.hkl

Additional supplementary materials:  crystallographic information; 3D view; checkCIF report

## Figures and Tables

**Table 1:** Hydrogen-bond geometry (Å, °)

*D*—H⋯*A*	*D*—H	H⋯*A*	*D*⋯*A*	*D*—H⋯*A*
O1*w*—H11⋯O5^i^	0.84 (1)	1.98 (2)	2.813 (6)	172 (7)
O1*w*—H12⋯O7^ii^	0.84 (1)	1.94 (2)	2.774 (6)	171 (8)
O2*w*—H21⋯O2	0.84 (1)	1.92 (2)	2.738 (7)	164 (7)
O2*w*—H22⋯O3*w*	0.84 (1)	1.84 (3)	2.65 (1)	162 (8)
O3*w*—H31⋯O7^iii^	0.84 (1)	2.03 (2)	2.80 (1)	152 (4)
O3*w*′—H33⋯O7^iii^	0.84 (1)	2.03 (2)	2.70 (2)	136 (3)
O4*w*—H41⋯O2^iv^	0.84 (1)	2.08 (3)	2.91 (1)	170 (13)
O4*w*—H42⋯O3*w*	0.84 (1)	1.98 (8)	2.65 (1)	136 (10)
O4*w*—H42⋯O3*w*′	0.84 (1)	1.99 (4)	2.79 (2)	159 (10)

Symmetry codes: (i) 
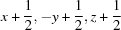
; (ii) 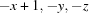; (iii) 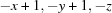; (iv) 
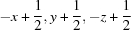
.

**Fig. 1.**
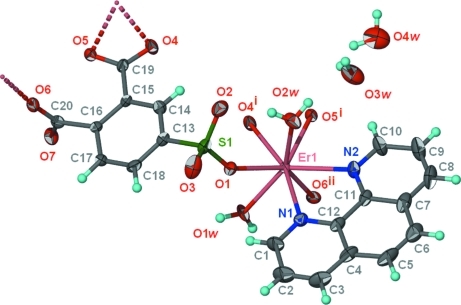
Thermal ellipsoid plot (Barbour, 2001) of the formula unit of polymeric [Er(H2O)2(C12H8N2)(C8H3O7S)]n.2nH2O at the 570% probability level; hydrogen atoms are drawn as spheres of arbitrary radius. The disorder is not shown.

**Fig. 2.**
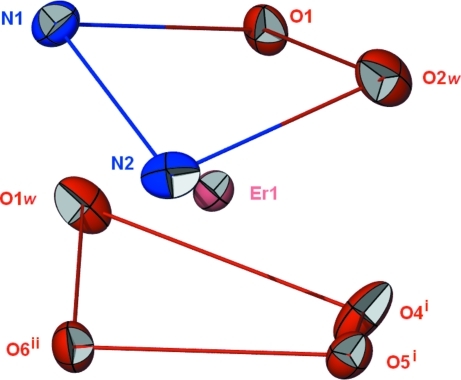
Square-antiprismatic geometry of Er.

## supplementary crystallographic information

### Comment

The deprotonated 4-sulfophthalic acid trianion forms a number of coordination polymers as its carboxyl and sulfo groups are capable of a variety of bonding modes. Among these, the 1,10-phenanthroline-coordinated europium derivative exists as a monoaqua coordination polymer adopting a chain motif (Xiao *et al.*, 2010). The title Er^III^ analog is instead a diaqua coordination polymer adopting a three-dimensional network motif. The 4-sulfophthalate trianion bridges three water/phenanthroline-coordinated Er^III^ atoms to form a three-dimensional network architecture (Scheme I, Fig. 1). The metal atom is chelated by a carboxyl group and is covalently bonded to a unidentate carboxyl as well as to a unidentate sulfo group in a square antiprismatic geometry (Fig. 2). The lattice water molecules are hydrogen-bonded to the network. Other O–H···O hydrogen bonds are also present (Table 1).

### Experimental

4-Sulfophthalic acid (0.080 g), 1,10-phenanthroline (0.057 g), erbium trichloride hexahydrate (0.114 g) and water (10 ml) were placed in a 25 -ml Teflon-lined stainless-steel Parr bomb. The vessel was heated at 443 K for 3 days. Faint pink crystals were obtained when the vessel was cooled to room temperature slowly in about 40% yield.

### Refinement

Carbon-bound H-atoms were placed in calculated positions (C—H 0.93 Å) and were included in the refinement in the riding model approximation, with *U*(H) set to 1.2*U*(C).

The water H-atoms were located in a difference Fourier map, and were refined with distance restraints of O–H 0.84±0.01 and H···H 1.37±0.01 Å; their temperature factors were tied by a factor of 1.5 times.

The O3w water molecule is disordered over tw sites in a 0.65 (3): 0.35 ratio. The disorder components share a common H atom, which forms a hydrogen bond to an acceptor atom.

The anisotropic temperature factors of the lattice water O atoms were tightly restrained to be nearly isotropic.

The final difference Fourier map had a peak at 0.64 Å from Er1 and a hole at 1.26 Å from this heavy atom.

### Crystal data

**Table:**

[Er(C_8_H_3_O_7_S)(C_12_H_8_N_2_)(H_2_O)_2_]·2H_2_O	*F*(000) = 1300
*M**_r_* = 662.69	*D*_x_ = 1.896 Mg m^−^^3^
Monoclinic, *P*2_1_/*n*	Mo *K*α radiation, λ = 0.71073 Å
Hall symbol: -P 2yn	Cell parameters from 5782 reflections
*a* = 14.3924 (1) Å	θ = 1.6–25.0°
*b* = 9.6206 (2) Å	µ = 3.77 mm^−^^1^
*c* = 17.4245 (3) Å	*T* = 293 K
β = 105.840 (1)°	Block, pink
*V* = 2321.04 (6) Å^3^	0.50 × 0.20 × 0.20 mm
*Z* = 4	

### Data collection

**Table:**

Bruker SMART APEX diffractometer	4014 independent reflections
Radiation source: fine-focus sealed tube	3799 reflections with *I* > 2σ(*I*)
graphite	*R*_int_ = 0.023
ω scans	θ_max_ = 25.0°, θ_min_ = 1.6°
Absorption correction: multi-scan (*SADABS*; Sheldrick, 1996)	*h* = −17→14
*T*_min_ = 0.500, *T*_max_ = 1.000	*k* = −10→11
7081 measured reflections	*l* = −18→20

### Refinement

**Table:**

Refinement on *F*^2^	Primary atom site location: structure-invariant direct methods
Least-squares matrix: full	Secondary atom site location: difference Fourier map
*R*[*F*^2^ > 2σ(*F*^2^)] = 0.037	Hydrogen site location: inferred from neighbouring sites
*wR*(*F*^2^) = 0.095	H atoms treated by a mixture of independent and constrained refinement
*S* = 1.09	*w* = 1/[σ^2^(*F*_o_^2^) + (0.0395*P*)^2^ + 16.686*P*] where *P* = (*F*_o_^2^ + 2*F*_c_^2^)/3
4014 reflections	(Δ/σ)_max_ = 0.001
353 parameters	Δρ_max_ = 0.97 e Å^−^^3^
33 restraints	Δρ_min_ = −1.21 e Å^−^^3^

### Fractional atomic coordinates and isotropic or equivalent isotropic displacement parameters (Å^2^)

**Table:**

	*x*	*y*	*z*	*U*_iso_*/*U*_eq_	Occ. (<1)
Er1	0.605750 (18)	0.47360 (3)	0.260342 (15)	0.02117 (11)	
S1	0.36918 (11)	0.32578 (18)	0.16175 (9)	0.0306 (3)	
O1	0.4701 (3)	0.3319 (5)	0.2100 (3)	0.0306 (10)	
O2	0.3281 (4)	0.4647 (6)	0.1432 (3)	0.0458 (13)	
O3	0.3114 (4)	0.2373 (7)	0.1977 (3)	0.0556 (15)	
O4	0.4072 (4)	0.4853 (5)	−0.1229 (3)	0.0418 (13)	
O5	0.3523 (3)	0.3144 (4)	−0.2054 (2)	0.0281 (9)	
O6	0.2667 (3)	0.0313 (4)	−0.1963 (3)	0.0316 (10)	
O7	0.4246 (3)	−0.0030 (5)	−0.1691 (3)	0.0376 (11)	
O1w	0.6548 (3)	0.2506 (5)	0.2388 (3)	0.0363 (11)	
H11	0.7135 (16)	0.234 (7)	0.260 (4)	0.054*	
H12	0.628 (4)	0.181 (5)	0.213 (4)	0.054*	
O2w	0.4707 (4)	0.6125 (5)	0.2474 (3)	0.0421 (12)	
H21	0.421 (4)	0.582 (7)	0.215 (4)	0.063*	
H22	0.471 (5)	0.6997 (12)	0.245 (5)	0.063*	
O3w	0.4830 (11)	0.8838 (11)	0.2752 (10)	0.067 (4)	0.65 (3)
H31	0.491 (5)	0.915 (11)	0.2327 (18)	0.100*	0.65 (3)
H32	0.524 (9)	0.918 (16)	0.314 (2)	0.100*	0.65 (3)
O3w'	0.4387 (18)	0.872 (2)	0.2221 (18)	0.062 (7)	0.35 (3)
H33	0.491 (5)	0.915 (11)	0.2327 (18)	0.093*	0.35 (3)
H34	0.421 (13)	0.86 (3)	0.173 (4)	0.093*	0.35 (3)
O4w	0.3479 (5)	1.0333 (8)	0.3134 (5)	0.077 (2)	
H41	0.293 (4)	1.015 (11)	0.320 (8)	0.116*	
H42	0.362 (8)	0.972 (9)	0.284 (7)	0.116*	
N1	0.5895 (4)	0.3629 (6)	0.3847 (3)	0.0305 (12)	
N2	0.6445 (4)	0.6325 (6)	0.3796 (3)	0.0333 (12)	
C1	0.5629 (6)	0.2322 (8)	0.3879 (4)	0.0421 (17)	
H1	0.5473	0.1810	0.3408	0.051*	
C2	0.5567 (7)	0.1661 (9)	0.4578 (5)	0.056 (2)	
H2	0.5377	0.0735	0.4568	0.067*	
C3	0.5786 (6)	0.2385 (10)	0.5265 (5)	0.053 (2)	
H3	0.5750	0.1959	0.5735	0.064*	
C4	0.6070 (5)	0.3794 (9)	0.5274 (4)	0.0421 (18)	
C5	0.6311 (6)	0.4650 (10)	0.5974 (4)	0.052 (2)	
H5	0.6265	0.4279	0.6456	0.063*	
C6	0.6601 (6)	0.5979 (10)	0.5955 (4)	0.051 (2)	
H6	0.6758	0.6503	0.6421	0.061*	
C7	0.6671 (5)	0.6591 (9)	0.5224 (4)	0.0440 (19)	
C8	0.6992 (6)	0.7949 (10)	0.5171 (5)	0.059 (2)	
H8	0.7183	0.8497	0.5627	0.070*	
C9	0.7025 (7)	0.8470 (9)	0.4456 (5)	0.061 (2)	
H9	0.7233	0.9376	0.4418	0.073*	
C10	0.6744 (6)	0.7634 (8)	0.3780 (5)	0.0468 (19)	
H10	0.6766	0.8008	0.3293	0.056*	
C11	0.6411 (5)	0.5800 (8)	0.4513 (4)	0.0315 (14)	
C12	0.6118 (4)	0.4375 (7)	0.4544 (4)	0.0310 (14)	
C13	0.3714 (4)	0.2483 (7)	0.0701 (3)	0.0275 (13)	
C14	0.3788 (4)	0.3306 (7)	0.0066 (4)	0.0288 (13)	
H14	0.3870	0.4262	0.0132	0.035*	
C15	0.3741 (4)	0.2703 (6)	−0.0666 (3)	0.0247 (12)	
C16	0.3647 (4)	0.1252 (6)	−0.0760 (3)	0.0238 (12)	
C17	0.3641 (5)	0.0436 (6)	−0.0099 (4)	0.0300 (14)	
H17	0.3625	−0.0528	−0.0144	0.036*	
C18	0.3657 (5)	0.1048 (7)	0.0627 (4)	0.0327 (15)	
H18	0.3630	0.0500	0.1060	0.039*	
C19	0.3780 (4)	0.3614 (7)	−0.1350 (3)	0.0263 (13)	
C20	0.3527 (4)	0.0479 (6)	−0.1535 (4)	0.0266 (13)	

### Atomic displacement parameters (Å^2^)

**Table:**

	*U*^11^	*U*^22^	*U*^33^	*U*^12^	*U*^13^	*U*^23^
Er1	0.02381 (16)	0.01893 (16)	0.02072 (16)	−0.00118 (10)	0.00599 (11)	−0.00058 (10)
S1	0.0253 (8)	0.0428 (9)	0.0233 (7)	−0.0038 (7)	0.0058 (6)	−0.0019 (7)
O1	0.024 (2)	0.029 (2)	0.032 (2)	−0.0041 (18)	−0.0026 (18)	−0.0005 (19)
O2	0.042 (3)	0.053 (3)	0.037 (3)	0.015 (2)	0.001 (2)	−0.006 (2)
O3	0.054 (3)	0.080 (4)	0.039 (3)	−0.030 (3)	0.023 (3)	−0.009 (3)
O4	0.076 (4)	0.023 (2)	0.026 (2)	−0.013 (2)	0.013 (2)	0.0003 (19)
O5	0.033 (2)	0.022 (2)	0.027 (2)	−0.0055 (18)	0.0060 (18)	0.0010 (17)
O6	0.027 (2)	0.027 (2)	0.037 (2)	−0.0018 (18)	0.0021 (19)	−0.0045 (19)
O7	0.027 (2)	0.036 (3)	0.048 (3)	0.003 (2)	0.007 (2)	−0.012 (2)
O1w	0.030 (2)	0.020 (2)	0.057 (3)	0.0006 (19)	0.008 (2)	−0.014 (2)
O2w	0.036 (3)	0.027 (3)	0.063 (3)	0.004 (2)	0.014 (2)	−0.002 (2)
O3w	0.082 (7)	0.046 (5)	0.081 (8)	0.004 (5)	0.040 (6)	0.004 (5)
O3w'	0.058 (10)	0.059 (9)	0.075 (11)	0.009 (7)	0.029 (8)	0.004 (8)
O4w	0.067 (4)	0.084 (5)	0.093 (5)	0.010 (4)	0.044 (4)	0.009 (4)
N1	0.033 (3)	0.033 (3)	0.026 (3)	−0.003 (2)	0.008 (2)	0.003 (2)
N2	0.038 (3)	0.035 (3)	0.030 (3)	−0.008 (2)	0.014 (2)	−0.008 (2)
C1	0.056 (5)	0.032 (4)	0.040 (4)	−0.005 (3)	0.017 (3)	0.010 (3)
C2	0.069 (6)	0.049 (5)	0.055 (5)	−0.006 (4)	0.025 (4)	0.022 (4)
C3	0.055 (5)	0.069 (6)	0.040 (4)	0.004 (4)	0.022 (4)	0.023 (4)
C4	0.036 (4)	0.065 (5)	0.024 (3)	0.005 (3)	0.008 (3)	0.008 (3)
C5	0.050 (5)	0.091 (7)	0.018 (3)	0.020 (5)	0.013 (3)	0.007 (4)
C6	0.051 (5)	0.070 (6)	0.032 (4)	0.014 (4)	0.011 (3)	−0.011 (4)
C7	0.031 (4)	0.065 (5)	0.035 (4)	0.003 (3)	0.008 (3)	−0.016 (4)
C8	0.058 (5)	0.069 (6)	0.047 (5)	−0.012 (4)	0.011 (4)	−0.030 (4)
C9	0.078 (6)	0.046 (5)	0.062 (6)	−0.019 (4)	0.026 (5)	−0.025 (4)
C10	0.062 (5)	0.033 (4)	0.052 (5)	−0.009 (4)	0.025 (4)	−0.012 (3)
C11	0.029 (3)	0.042 (4)	0.024 (3)	−0.002 (3)	0.008 (3)	−0.007 (3)
C12	0.028 (3)	0.045 (4)	0.021 (3)	0.004 (3)	0.008 (3)	0.001 (3)
C13	0.024 (3)	0.035 (3)	0.022 (3)	−0.001 (3)	0.003 (2)	−0.001 (3)
C14	0.029 (3)	0.027 (3)	0.031 (3)	−0.002 (3)	0.009 (3)	0.000 (3)
C15	0.025 (3)	0.026 (3)	0.019 (3)	0.001 (2)	0.001 (2)	−0.001 (2)
C16	0.020 (3)	0.025 (3)	0.023 (3)	0.003 (2)	0.001 (2)	0.002 (2)
C17	0.036 (3)	0.022 (3)	0.028 (3)	0.001 (3)	0.001 (3)	0.000 (2)
C18	0.032 (3)	0.037 (4)	0.025 (3)	−0.003 (3)	0.000 (3)	0.009 (3)
C19	0.029 (3)	0.029 (3)	0.019 (3)	0.000 (3)	0.005 (2)	−0.004 (2)
C20	0.023 (3)	0.024 (3)	0.033 (3)	−0.002 (2)	0.008 (3)	0.002 (3)

### Geometric parameters (Å, °)

**Table:**

Er1—O6^i^	2.233 (4)	N2—C10	1.334 (9)
Er1—O2w	2.319 (5)	N2—C11	1.362 (8)
Er1—O1w	2.321 (4)	C1—C2	1.399 (10)
Er1—O1	2.346 (4)	C1—H1	0.9300
Er1—O4^ii^	2.384 (5)	C2—C3	1.346 (12)
Er1—O5^ii^	2.399 (4)	C2—H2	0.9300
Er1—N1	2.483 (5)	C3—C4	1.414 (12)
Er1—N2	2.516 (5)	C3—H3	0.9300
S1—O3	1.447 (5)	C4—C12	1.409 (9)
S1—O2	1.462 (5)	C4—C5	1.434 (11)
S1—O1	1.468 (4)	C5—C6	1.348 (13)
S1—C13	1.771 (6)	C5—H5	0.9300
O4—C19	1.263 (8)	C6—C7	1.430 (11)
O4—Er1^ii^	2.384 (5)	C6—H6	0.9300
O5—C19	1.264 (7)	C7—C8	1.398 (13)
O5—Er1^ii^	2.399 (4)	C7—C11	1.415 (9)
O6—C20	1.268 (8)	C8—C9	1.356 (13)
O6—Er1^iii^	2.233 (4)	C8—H8	0.9300
O7—C20	1.240 (8)	C9—C10	1.392 (11)
O1w—H11	0.84 (1)	C9—H9	0.9300
O1w—H12	0.84 (1)	C10—H10	0.9300
O2w—H21	0.84 (1)	C11—C12	1.439 (10)
O2w—H22	0.84 (1)	C13—C18	1.386 (9)
O3w—H31	0.84 (1)	C13—C14	1.388 (9)
O3w—H32	0.84 (1)	C14—C15	1.385 (8)
O3w—H33	0.84 (1)	C14—H14	0.9300
O3w'—H31	0.84 (1)	C15—C16	1.408 (9)
O3w'—H33	0.84 (1)	C15—C19	1.494 (8)
O3w'—H34	0.84 (1)	C16—C17	1.397 (9)
O4w—H41	0.84 (1)	C16—C20	1.510 (8)
O4w—H42	0.84 (1)	C17—C18	1.390 (9)
N1—C1	1.320 (9)	C17—H17	0.9300
N1—C12	1.371 (8)	C18—H18	0.9300
			
O6^i^—Er1—O2w	144.10 (17)	C3—C2—C1	118.9 (8)
O6^i^—Er1—O1w	72.66 (16)	C3—C2—H2	120.5
O2w—Er1—O1w	143.22 (17)	C1—C2—H2	120.5
O6^i^—Er1—O1	142.63 (15)	C2—C3—C4	120.2 (7)
O2w—Er1—O1	73.08 (17)	C2—C3—H3	119.9
O1w—Er1—O1	70.17 (16)	C4—C3—H3	119.9
O6^i^—Er1—O4^ii^	97.62 (19)	C12—C4—C3	117.1 (7)
O2w—Er1—O4^ii^	88.5 (2)	C12—C4—C5	118.7 (8)
O1w—Er1—O4^ii^	86.24 (18)	C3—C4—C5	124.2 (7)
O1—Er1—O4^ii^	83.77 (16)	C6—C5—C4	121.9 (7)
O6^i^—Er1—O5^ii^	78.60 (15)	C6—C5—H5	119.1
O2w—Er1—O5^ii^	76.50 (17)	C4—C5—H5	119.1
O1w—Er1—O5^ii^	126.94 (16)	C5—C6—C7	120.7 (7)
O1—Er1—O5^ii^	128.25 (14)	C5—C6—H6	119.7
O4^ii^—Er1—O5^ii^	54.39 (15)	C7—C6—H6	119.7
O6^i^—Er1—N1	91.84 (17)	C8—C7—C11	117.3 (7)
O2w—Er1—N1	93.20 (19)	C8—C7—C6	123.2 (7)
O1w—Er1—N1	81.37 (18)	C11—C7—C6	119.5 (8)
O1—Er1—N1	79.05 (16)	C9—C8—C7	120.1 (7)
O4^ii^—Er1—N1	161.43 (17)	C9—C8—H8	120.0
O5^ii^—Er1—N1	143.79 (16)	C7—C8—H8	120.0
O6^i^—Er1—N2	75.99 (17)	C8—C9—C10	119.3 (8)
O2w—Er1—N2	73.81 (19)	C8—C9—H9	120.4
O1w—Er1—N2	133.36 (19)	C10—C9—H9	120.4
O1—Er1—N2	129.52 (16)	N2—C10—C9	123.3 (8)
O4^ii^—Er1—N2	131.89 (17)	N2—C10—H10	118.3
O5^ii^—Er1—N2	77.84 (16)	C9—C10—H10	118.3
N1—Er1—N2	65.95 (18)	N2—C11—C7	122.5 (7)
O3—S1—O2	112.9 (4)	N2—C11—C12	118.2 (5)
O3—S1—O1	111.8 (3)	C7—C11—C12	119.3 (6)
O2—S1—O1	111.6 (3)	N1—C12—C4	122.3 (7)
O3—S1—C13	107.3 (3)	N1—C12—C11	117.8 (5)
O2—S1—C13	106.9 (3)	C4—C12—C11	119.9 (6)
O1—S1—C13	105.9 (3)	C18—C13—C14	120.7 (6)
S1—O1—Er1	146.1 (3)	C18—C13—S1	119.1 (5)
C19—O4—Er1^ii^	93.2 (4)	C14—C13—S1	120.2 (5)
C19—O5—Er1^ii^	92.5 (4)	C15—C14—C13	119.9 (6)
C20—O6—Er1^iii^	163.2 (4)	C15—C14—H14	120.0
Er1—O1w—H11	115 (4)	C13—C14—H14	120.0
Er1—O1w—H12	136 (4)	C14—C15—C16	120.1 (6)
H11—O1w—H12	110 (2)	C14—C15—C19	119.1 (5)
Er1—O2w—H21	114 (6)	C16—C15—C19	120.8 (5)
Er1—O2w—H22	125 (6)	C17—C16—C15	118.9 (5)
H21—O2w—H22	109 (2)	C17—C16—C20	115.9 (5)
H31—O3w—H32	110 (2)	C15—C16—C20	125.2 (5)
H32—O3w—H33	110 (2)	C18—C17—C16	120.7 (6)
H31—O3w'—H34	109 (2)	C18—C17—H17	119.6
H33—O3w'—H34	109 (2)	C16—C17—H17	119.6
H41—O4w—H42	109 (2)	C13—C18—C17	119.4 (6)
C1—N1—C12	117.6 (6)	C13—C18—H18	120.3
C1—N1—Er1	122.9 (5)	C17—C18—H18	120.3
C12—N1—Er1	119.5 (4)	O4—C19—O5	119.8 (6)
C10—N2—C11	117.5 (6)	O4—C19—C15	120.2 (5)
C10—N2—Er1	123.9 (5)	O5—C19—C15	120.0 (5)
C11—N2—Er1	118.5 (4)	O7—C20—O6	124.1 (6)
N1—C1—C2	123.9 (7)	O7—C20—C16	119.4 (5)
N1—C1—H1	118.0	O6—C20—C16	116.3 (5)
C2—C1—H1	118.0		
			
O3—S1—O1—Er1	−138.7 (5)	C8—C9—C10—N2	−0.5 (14)
O2—S1—O1—Er1	−11.2 (6)	C10—N2—C11—C7	0.5 (10)
C13—S1—O1—Er1	104.8 (5)	Er1—N2—C11—C7	176.3 (5)
O6^i^—Er1—O1—S1	−155.0 (4)	C10—N2—C11—C12	−178.1 (6)
O2w—Er1—O1—S1	29.7 (5)	Er1—N2—C11—C12	−2.3 (8)
O1w—Er1—O1—S1	−148.9 (5)	C8—C7—C11—N2	−1.4 (11)
O4^ii^—Er1—O1—S1	−60.6 (5)	C6—C7—C11—N2	178.9 (6)
O5^ii^—Er1—O1—S1	−26.9 (6)	C8—C7—C11—C12	177.2 (7)
N1—Er1—O1—S1	126.5 (5)	C6—C7—C11—C12	−2.5 (10)
N2—Er1—O1—S1	80.7 (5)	C1—N1—C12—C4	−0.1 (10)
O6^i^—Er1—N1—C1	−106.2 (6)	Er1—N1—C12—C4	−178.0 (5)
O2w—Er1—N1—C1	109.3 (6)	C1—N1—C12—C11	179.9 (6)
O1w—Er1—N1—C1	−34.1 (5)	Er1—N1—C12—C11	2.0 (7)
O1—Er1—N1—C1	37.3 (5)	C3—C4—C12—N1	0.5 (10)
O4^ii^—Er1—N1—C1	14.6 (9)	C5—C4—C12—N1	−179.6 (6)
O5^ii^—Er1—N1—C1	−179.3 (5)	C3—C4—C12—C11	−179.5 (6)
N2—Er1—N1—C1	−180.0 (6)	C5—C4—C12—C11	0.4 (10)
O6^i^—Er1—N1—C12	71.5 (5)	N2—C11—C12—N1	0.3 (9)
O2w—Er1—N1—C12	−72.9 (5)	C7—C11—C12—N1	−178.4 (6)
O1w—Er1—N1—C12	143.7 (5)	N2—C11—C12—C4	−179.8 (6)
O1—Er1—N1—C12	−145.0 (5)	C7—C11—C12—C4	1.6 (9)
O4^ii^—Er1—N1—C12	−167.6 (5)	O3—S1—C13—C18	−30.7 (6)
O5^ii^—Er1—N1—C12	−1.6 (6)	O2—S1—C13—C18	−152.0 (5)
N2—Er1—N1—C12	−2.2 (4)	O1—S1—C13—C18	88.9 (5)
O6^i^—Er1—N2—C10	79.3 (6)	O3—S1—C13—C14	149.3 (5)
O2w—Er1—N2—C10	−81.0 (6)	O2—S1—C13—C14	28.0 (6)
O1w—Er1—N2—C10	128.2 (6)	O1—S1—C13—C14	−91.1 (5)
O1—Er1—N2—C10	−131.8 (6)	C18—C13—C14—C15	4.3 (9)
O4^ii^—Er1—N2—C10	−8.4 (7)	S1—C13—C14—C15	−175.7 (5)
O5^ii^—Er1—N2—C10	−1.8 (6)	C13—C14—C15—C16	−1.9 (9)
N1—Er1—N2—C10	177.8 (6)	C13—C14—C15—C19	177.4 (6)
O6^i^—Er1—N2—C11	−96.1 (5)	C14—C15—C16—C17	−2.5 (9)
O2w—Er1—N2—C11	103.5 (5)	C19—C15—C16—C17	178.2 (6)
O1w—Er1—N2—C11	−47.3 (6)	C14—C15—C16—C20	176.1 (5)
O1—Er1—N2—C11	52.7 (5)	C19—C15—C16—C20	−3.2 (9)
O4^ii^—Er1—N2—C11	176.2 (4)	C15—C16—C17—C18	4.6 (9)
O5^ii^—Er1—N2—C11	−177.2 (5)	C20—C16—C17—C18	−174.1 (6)
N1—Er1—N2—C11	2.3 (4)	C14—C13—C18—C17	−2.2 (10)
C12—N1—C1—C2	−0.3 (11)	S1—C13—C18—C17	177.8 (5)
Er1—N1—C1—C2	177.5 (6)	C16—C17—C18—C13	−2.3 (10)
N1—C1—C2—C3	0.2 (13)	Er1^ii^—O4—C19—O5	−2.9 (6)
C1—C2—C3—C4	0.2 (13)	Er1^ii^—O4—C19—C15	176.8 (5)
C2—C3—C4—C12	−0.5 (12)	Er1^ii^—O5—C19—O4	2.8 (6)
C2—C3—C4—C5	179.6 (8)	Er1^ii^—O5—C19—C15	−176.9 (5)
C12—C4—C5—C6	−1.6 (11)	C14—C15—C19—O4	16.2 (9)
C3—C4—C5—C6	178.3 (8)	C16—C15—C19—O4	−164.5 (6)
C4—C5—C6—C7	0.6 (12)	C14—C15—C19—O5	−164.1 (6)
C5—C6—C7—C8	−178.2 (8)	C16—C15—C19—O5	15.2 (9)
C5—C6—C7—C11	1.4 (11)	Er1^iii^—O6—C20—O7	165.7 (11)
C11—C7—C8—C9	1.4 (12)	Er1^iii^—O6—C20—C16	−9.2 (18)
C6—C7—C8—C9	−178.9 (8)	C17—C16—C20—O7	−85.4 (7)
C7—C8—C9—C10	−0.5 (14)	C15—C16—C20—O7	96.0 (8)
C11—N2—C10—C9	0.5 (12)	C17—C16—C20—O6	89.7 (7)
Er1—N2—C10—C9	−175.0 (7)	C15—C16—C20—O6	−88.8 (7)

Symmetry codes: (i) *x*+1/2, −*y*+1/2, *z*+1/2; (ii) −*x*+1, −*y*+1, −*z*; (iii) *x*−1/2, −*y*+1/2, *z*−1/2.

### Hydrogen-bond geometry (Å, °)

**Table:**

*D*—H···*A*	*D*—H	H···*A*	*D*···*A*	*D*—H···*A*
O1w—H11···O5^i^	0.84 (1)	1.98 (2)	2.813 (6)	172 (7)
O1w—H12···O7^iv^	0.84 (1)	1.94 (2)	2.774 (6)	171 (8)
O2w—H21···O2	0.84 (1)	1.92 (2)	2.738 (7)	164 (7)
O2w—H22···O3w	0.84 (1)	1.84 (3)	2.65 (1)	162 (8)
O3w—H31···O7^ii^	0.84 (1)	2.03 (2)	2.80 (1)	152 (4)
O3w'—H33···O7^ii^	0.84 (1)	2.03 (2)	2.70 (2)	136 (3)
O4w—H41···O2^v^	0.84 (1)	2.08 (3)	2.91 (1)	170 (13)
O4w—H42···O3w	0.84 (1)	1.98 (8)	2.65 (1)	136 (10)
O4w—H42···O3w'	0.84 (1)	1.99 (4)	2.79 (2)	159 (10)

Symmetry codes: (i) *x*+1/2, −*y*+1/2, *z*+1/2; (iv) −*x*+1, −*y*, −*z*; (ii) −*x*+1, −*y*+1, −*z*; (v) −*x*+1/2, *y*+1/2, −*z*+1/2.

## References

[bb1] Barbour, L. J. (2001). *J. Supramol. Chem.* **1**, 189–191.

[bb2] Bruker (2005). *APEX2* and *SAINT* Bruker AXS Inc., Madison, Wisconsin, USA.

[bb3] Sheldrick, G. M. (1996). *SADABS* University of Göttingen, Germany.

[bb4] Sheldrick, G. M. (2008). *Acta Cryst.* A**64**, 112–122. 10.1107/S0108767307043930 18156677

[bb5] Westrip, S. P. (2010). *J. Appl. Cryst.* **43**, 920–925.

[bb6] Xiao, S.-S., Zheng, X.-J., Yan, S.-H. & Deng, X.-B. (2010). *CrystEngComm*, **12**, 3145–3151.

